# Improving the Shape Memory Effect of a Fe-Mn-Si-Cr-Ni Alloy through Shot Peening

**DOI:** 10.3390/ma15072585

**Published:** 2022-03-31

**Authors:** Huanping Yang, Wenbin Yan, Xuyang Deng, Mengqi Zhang, Yaomian Wang

**Affiliations:** 1 School of Metallurgical Engineering, Xi’an University of Architecture and Technology, Xi’an 710055, China; yanwenbin0609@163.com (W.Y.); 13571823102@163.com (X.D.); zhangmengqi113@163.com (M.Z.); 2 Shaanxi Key Laboratory of Nano Materials and Technology, Xi’an 710055, China

**Keywords:** shape memory alloy, Fe-Mn-Si-Cr-Ni alloy, shot peening, microstructure, phase transformation

## Abstract

To improve the shape memory effect, the solutionized Fe-24Mn-6Si-9Cr-6Ni alloy was shot peened and subsequently annealed. The phase constituent was examined using the X-ray diffraction method. Microstructure evolution was characterized using an optical microscope and the electronic backscatter diffraction method, and the shape memory effect was evaluated using a bending test. The results show that α′-martensite and ε-martensite were introduced into the shot-peened surface layer. The α′-martensite remained after annealing even at 850 °C. Microstructure of the surface layer was refined through shot peening and subsequent annealing. Compared with those of the solutionized specimen, the shape recovery ratio and recovery strain of the specimens that are shot peened and subsequently annealed are significantly improved at different prestrains.

## 1. Introduction

Shape memory alloys (SMAs) are a type of material that can restore their original shape after deformation. NiTi alloys are excellent SMAs. They can recover large strains of 6–8% due to the shape memory effect and superelasticity [1,2]. However, they exhibit low cold workability and high cost, limiting their large-scale applications [1,3]. Consequently, Fe-Mn-Si-based SMAs have attracted significant interest recently because of their comparable shape memory effect and low cost [3,4,5,6,7,8].

The shape memory effect of Fe-Mn-Si-based SMAs results from a stress-induced γ-austenite to ε-martensite phase transformation and its reverse transformation on subsequent heating above the temperature *A_f_*. Shear displacement causes the martensitic transformation, resulting in the formation of Shockley partial dislocations and stacking faults on (111)_γ_ close-packed atomic planes. There are 12 shear systems consisting of four (111) planes and three <112> directions. Therefore, 12 variants of the ε-martensite can be formed when the γ-austenite is loaded. Only one variant of ε-martensite was introduced into single crystal Fe-Mn-Si alloys using tensile stress applied along the <414> direction, yielding a large recovery strain of 9% [9]. Collisions between the different variants occurred as the number of variants increased, and an α′-martensite was produced, decreasing the shape recovery [10,11,12]. Efforts should be made to promote martensitic transformation on plastic deformation and the crystallographic reversibility of the reverse transformation to achieve a high shape memory effect [13].

For the polycrystalline Fe-Mn-Si-based SMAs, a good shape memory effect is more likely if the alloy has low stacking-fault energy, high strength of the parent phase, *M_s_* near Néel temperature *T_N_*, and an ideal *c*/*a* ratio (1.633) of the ε phase [14]. Chemical composition design can influence the shape memory effect by affecting the parent matrix mechanical property, phase stability, lattice parameters, stacking-fault energy, *M_s,_* and *T_N_* [13]. Mn and Si constituents are the necessary elements in Fe-Mn-Si-based SMAs. Mn can harden the γ-austenite and increase the reversibility of ε-martensite by inhibiting the formation of α′-martensite [15]. Si enhances reversibility by reducing the transformation volume change and interfacial atomic mismatch [16]. Adding Cr and Ni could increase the corrosion resistance and improve the shape memory effect by increasing the *c*/*a* ratio [17].

Except for the alloy design, different processing technologies have been used to improve shape memory effect by optimizing the microstructure [1]. It has been indicated that second-phase precipitates in Fe-15Mn-5Si-9Cr-5Ni SMA can be produced via conventional rolling, asymmetric rolling, and equal-channel angular (ECAP) pressing followed by annealing [7]. Ultrafine or fine grains were induced in Fe-Mn-Si-based SMAs via high-ratio differential speed rolling [18], ECAP [19], and high-speed high-pressure torsion [20], and their shape memory effect was improved significantly. Shot peening is a universal surface modification process used for many engineering equipment and parts, that can refine the microstructure, strengthen the material, and introduce residual stress. Severe plastic deformation happens in the surface layer and gradually decreases with depth for the material subjected to shot peening [21]. Therefore, significant microstructure variation can be expected in the surface layer of the shot-peened SMAs.

This study explores the feasibility of improving the shape memory effect of Fe-Mn-Si-Cr-Ni alloy using shot peening and examines the microstructural evolution. In this study, a Fe-Mn-Si-Cr-Ni alloy was shot peened and subsequently annealed. The phase constitution and microstructural evolution were investigated. The shape memory effect was evaluated using a bending test, and the influence of shot peening on the shape memory effect was discussed.

## 2. Experimental Details

The Fe-24Mn-6Si-9Cr-6Ni alloy was produced through a process consisting of vacuum melting, casting, forging, and hot rolling. The chemical composition of the alloy was analyzed using a spectrometer (Perkin Elmer Optima 8300, Waltham, MA, USA), and the results are shown in Table 1.

The solution treatment was conducted at 950 °C for 1 h using a Muffle furnace, followed by a water quenching. Subsequently, the solutionized alloy was shot peened. The peening parameters are summarized in Table 2. The shot-peened alloy was annealed to improve the plasticity. Three different holding temperatures (650 °C, 750 °C, and 850 °C) were used for annealing for 30 min.

**Figure 1:**
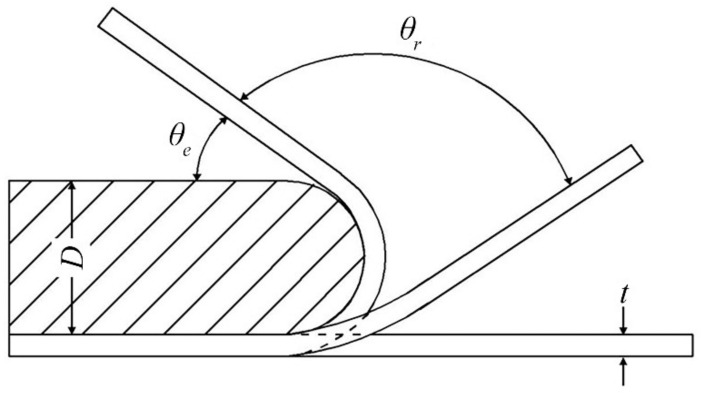
Schematic illustration of bending test for shape memory effect.

Before measuring the shape memory effect, the specimens were cleaned in a solution (5 mL HNO_3_ + 10 mL H_2_O_2_ + 20 mL HCl + 70 mL H_2_O) to remove the oxidation layer. The shape memory effect was evaluated using a conventional bending method (Figure 1). The shot-peened surface was compressed during the bending test. The 0.7-mm-thick specimens were bent to $\theta_{0}=180{}^{\circ}$ and maintained for 15 s using molds of different diameters at room temperature. The maximum tensile/compressive strain that occurred in the outer/inner surface was taken as the prestrain ε and is expressed using the following:

(1) $$\varepsilon =\frac{t}{D+t}$$

where *D* is the bend diameter and *t* is the specimen thickness. The bent specimens recovered elastically with angles of $\theta_{e}$ when the loading was removed. Then, the specimens were heated at 600 °C for 15 min. The deformed shape was partly recovered, and the recovery angles $\theta_{r}$ were measured when the specimens were cooled to room temperature. The shape recovery ratio η was determined using the following:

(2) $$\eta =\frac{\theta_{r}}{\theta_{0}-\theta_{e}}$$

The corresponding recovery strain $\varepsilon_{r}\;$was given by

(3) $$\varepsilon_{r}=\frac{\theta_{r}}{\theta_{0}}\varepsilon$$

Specimens for optical microstructure observation were prepared through mechanical polishing and chemical etching with a solution of hydrofluoric acid, hydrochloric acid, and pure water in 1:10:30 volume ratio. The observation was performed on an inverted metallurgical microscope using polarized light. Specimens for the electronic backscatter diffraction (EBSD) analysis were electropolished in an electrolytic solution of perchloric acid and ethyl alcohol with a 1:9 volume ratio using a voltage of 20 V for 22 s. The EBSD measurements were performed in a field emission scanning electron microscope (Gemini 300, Oberkochen, Germany) equipped with a Nordlys Nano EBSD detector at 20 kV and a step size of 0.2 μm.

X-ray diffraction (XRD) analysis was performed on a diffractometer (Bruker D8 ADVANCE, Billerica, United States) with Cu Kα radiation. The tube voltage and current were 40 kV and 40 mA, respectively. Scans were made in the 2θ range from 35° to 105° with a 0.02° step size and 0.1°/s scanning speed.

## 3. Results and Discussion

### 3.1. Phase Analysis

In the Fe-Mn-Si-Cr-Ni SMAs, γ-austenite, ε-martensite, and α′-martensite are three major phases. The lattice parameters of these phases vary with composition and manufacturing route [7,22,23,24], indicating that the diffraction angle 2θ of a certain crystal plane can shift in a range. The crystal structure and ranges of the lattice parameters are summarized in Table 3. According to the lattice parameters, diffraction angles were calculated using Bragg’s law. The phases can be identified using the XRD pattern.

**Figure 2:**
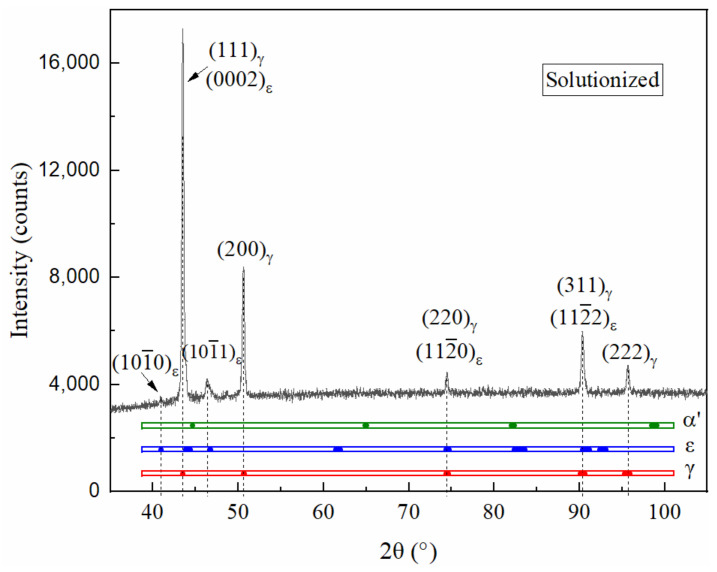
XRD pattern of the solutionized specimen.

Figure 2 shows the XRD pattern of the specimen solutionized at 950 °C for 1 h. The 2θ distribution ranges of the three phases’ different crystal planes are also illustrated using the different blocks in the three bands to help identify the phases. The alloy is constituted by γ-austenite and ε-martensite after the solution treatment.

**Figure 3:**
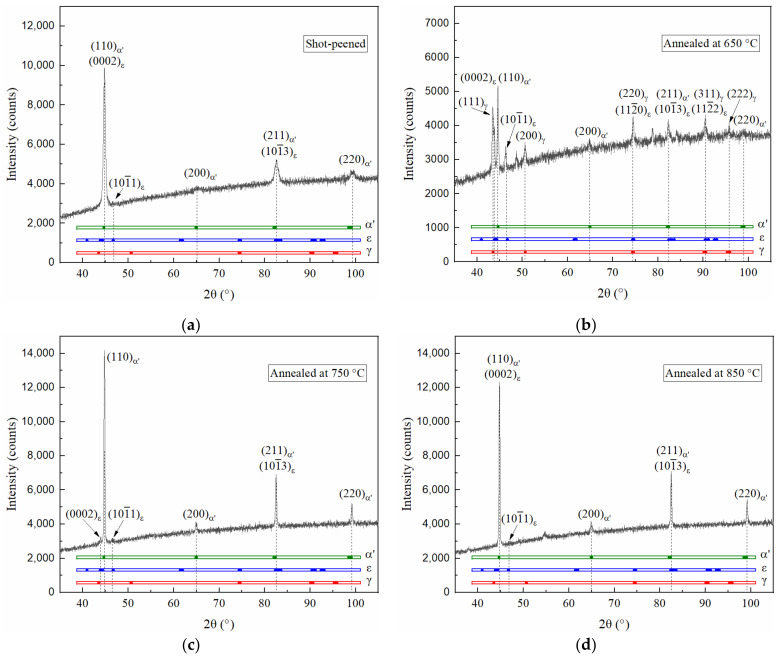
XRD patterns of (**a**) the shot-peened specimen and the subsequently annealed specimens with annealing temperatures of (**b**) 650 °C, (**c**) 750 °C, and (**d**) 850 °C.

After the solution treatment, the Fe-24Mn-6Si-9Cr-6Ni alloy was shot peened. The XRD pattern of the peened surface layer is shown in Figure 3. The diffraction peak of (110)_α′_ overlaps with that of (0002)_ε_ at 2θ = 44.7674°, and (211)_α′_ overlaps with (10$\bar{1}$3)_ε_ at 2θ = 82.6083°, as shown in Figure 3a. The presence of ε-martensite and α′-martensite cannot be inferred from these two peaks. Nevertheless, significant diffraction peaks of (200)_α′_ and (220)_α′_ can be observed at 65.0844° and 99.3183°, respectively. A weak peak of (10$\bar{1}$1)_ε_ is present at 46.8263°. Hence, it can be confirmed that the surface layer of the shot-peened Fe-24Mn-6Si-9Cr-6Ni alloy consists of α′-martensite and ε-martensite. This experimental result is unexpected because Mn can prevent the formation of α′-martensite [15], which is usually introduced in the Fe-Mn-Si alloys with < 20 wt% Mn content, such as Fe-14Mn-5Si-8Cr-4Ni [25], Fe–14Mn–5Si–9Cr–5Ni [26,27], and Fe-18Mn-5.5Si-9.5Cr-Ni [12].

After shot peening, the specimens were annealed at 650 °C, 750 °C, and 850 °C for 30 min. The γ-austenite, ε-martensite, and α′-martensite can be identified for the specimen annealed at 650 °C from the XRD pattern shown in Figure 3b, indicating that a substantial reverse martensitic transformation occurred during annealing at 650 °C for the shot-peened specimen. However, the diffraction peaks of γ-austenite cannot be observed as the annealing temperature increases to 750 °C. The ε-martensite peaks also become weak. Compared with the (200)_α′_ and (220)_α′_ diffraction peaks shown in Figure 3a,b, the two peaks’ intensity increased and the their full width at half maximum decreased as the annealing temperature increased to 750 °C. When the annealing temperature was increased to 850 °C, the (10$\bar{1}$1)_ε_ diffraction peak almost disappeared and the (200)_α′_ and (220)_α′_ peak intensities increased by 24.4% and 17.2%, respectively, compared with those of the specimen annealed at 750 °C. This implies that the volume fraction of α′-martensite in the shot-peened surface layer increased when the annealing temperature increased from 650 °C to 850 °C.

### 3.2. Microstructural Evolution

**Figure 4:**
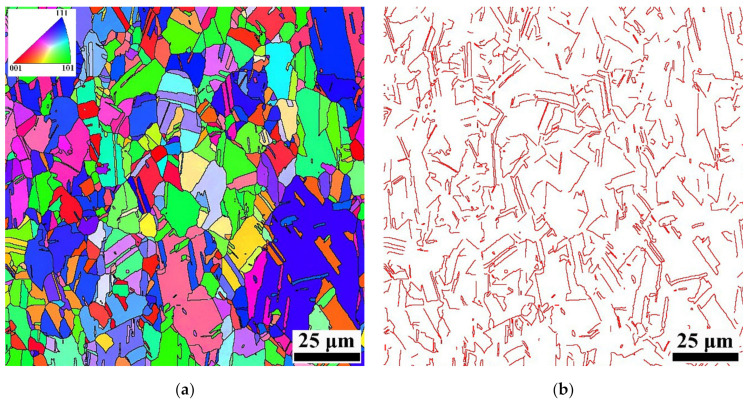
EBSD images of the solutionized specimen: (**a**) inverse pole figure superimposed with grain boundaries and (**b**) map of ∑3 twin boundaries.

Figure 4 shows the EBSD images of the solutionized specimen, which shows that the microstructure is fine. The mean grain size is estimated to be approximately 9 μm by analyzing Figure 4a. It has been pointed out that twins are commonly observed in γ-austenite of Fe-Mn-Si-based SMAs after thermo-mechanical processing [3,8]. In this study, a high density of twin boundary can be found in the solutionized Fe-24Mn-6Si-9Cr-6Ni alloy (Figure 4b). Investigations have shown that twin boundaries are harmful to the shape memory effect of Fe-Mn-Si-based SMAs because of the interactions between the twin boundary and ε-martensite [3,8].

**Figure 5:**
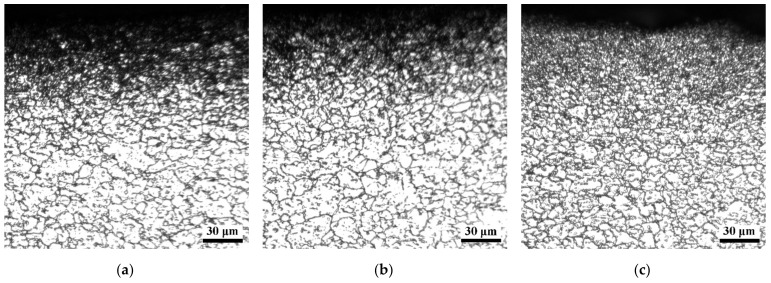
Cross-sectional optical microstructure of the shot-peened and the subsequently annealed specimens with (**a**) 650 °C, (**b**) 750 °C, and (**c**) 850 °C annealing temperatures.

**Figure 6:**
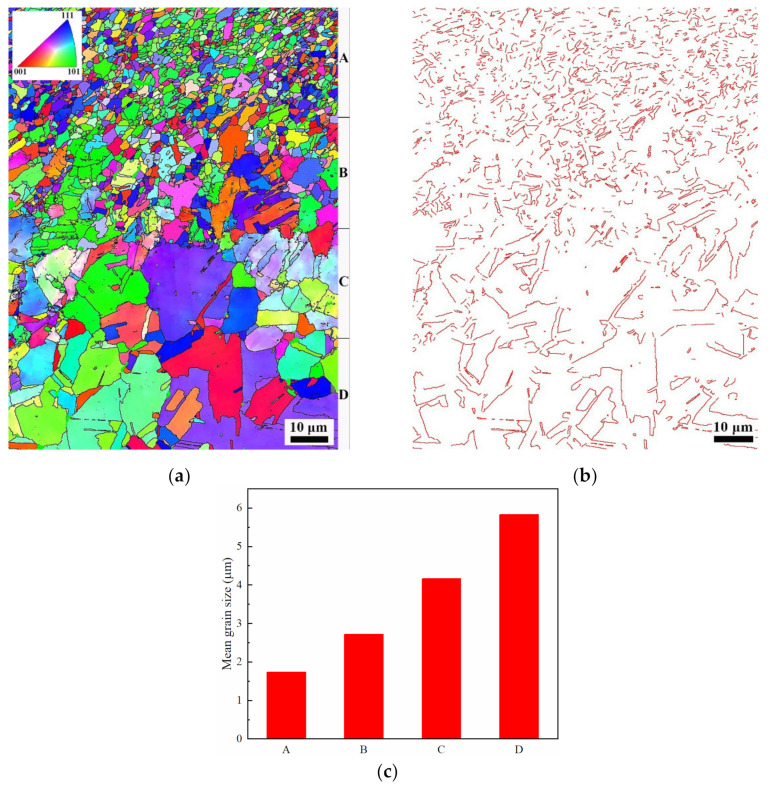
EBSD images of the specimen shot peened and subsequently annealed at 850 °C: (**a**) inverse pole figure superimposed with grain boundaries, (**b**) map of ∑3 twin boundaries, and (**c**) mean grain size of the different layers indicated in (**a**).

As a surface severe plastic deformation processing technology, shot peening can introduce high dislocation density and stored energy in the surface layer of the target materials. A significant microstructural evolution of the surface layer can happen during subsequent annealing at high temperature. For the shot-peened Fe-24Mn-6Si-9Cr-6Ni alloy annealed at 650 °C in this study, Figure 5a shows that the morphology of the surface layer exhibits marked difference from the matrix, which can be attributed to the occurrence of reverse martensitic transformation, recovery, and recrystallization in the surface layer during annealing. This indicates refinement of the microstructure results from the shot peening and subsequent annealing. As the annealing temperature increased, the amount of recrystallization of the surface layer increased (Figure 5a,c). Small-sized recrystallized grains can be seen in the surface layer of the specimen annealed at 850 °C. EBSD images of the surface layer of the specimen annealed at 850 °C are shown in Figure 6a,b. Because of the difference in recrystallization behavior, the grains of layers A, B, C, and D gradually become larger as the depth from the shot-peened surface increases. The mean grain size of the top surface layer A with a depth of approximately 30 μm is approximately 1.7 μm, which is much smaller than that of the solutionized specimen. The mean grain size of layer B increased to approximately 2.7 μm. There are several grains with large sizes in layers C and D, but the mean grain size remains small. The variation of mean grain sizes across the four layers is illustrated in Figure 6c, showing a significant gradual distribution. Meanwhile, many twin boundaries can be found in the specimen shot peened and subsequently annealed at 850 °C (Figure 6a). The corresponding map of ∑3 twin boundaries is shown in Figure 6b, showing that the density of twin boundaries is significantly high in the surface layer and decreases gradually with depth. The density of twin boundaries in the matrix is comparable to that of the solutionized specimen shown in Figure 4b.

### 3.3. Property

**Figure 7:**
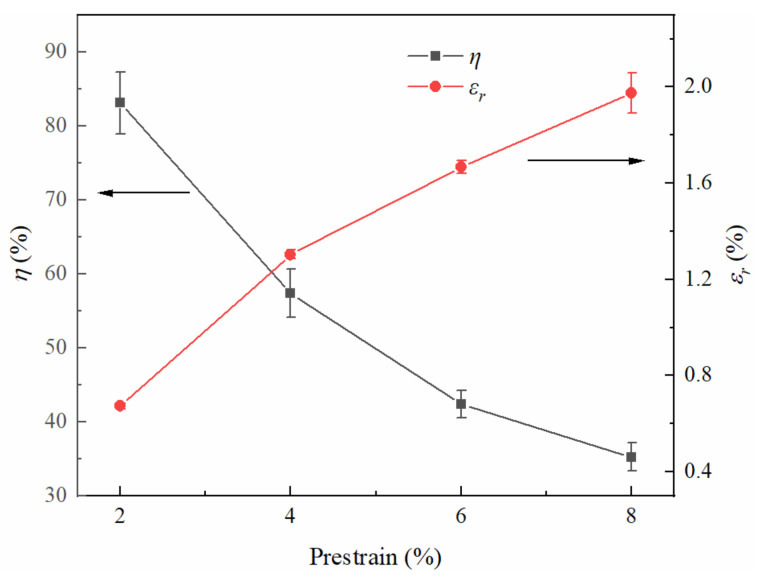
Shape memory effect of the solutionized specimens.

Figure 7 shows the shape recovery ratio and recovery strain of the solutionized specimen. The shape recovery ratio decreased from 83.1% to 35.2% when the prestrain increased from 2% to 8% and the corresponding recovery strain increased from 0.67% to 1.97%.

**Figure 8:**
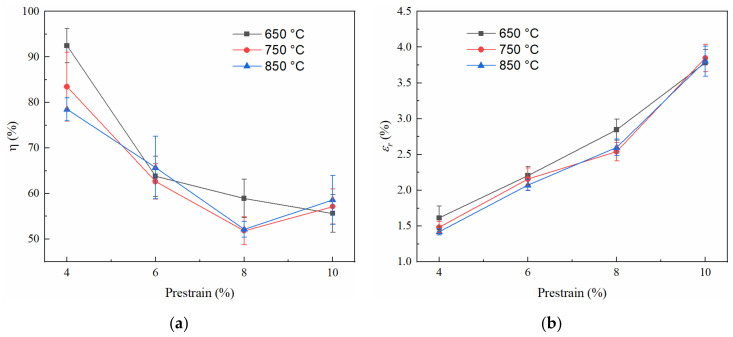
Shape memory effect of specimens subjected to shot peening and subsequent annealing at 650 °C, 750 °C, and 850 °C temperatures: (**a**) shape recovery ratio and (**b**) recovery strain.

The shape memory effect after shot peening and subsequent annealing at different temperatures is shown in Figure 8. The shape recovery ratio is shown to be 78.5% at 4% prestrain for the specimen annealed at 850 °C, and it can be increased to 92.5% with a 650 °C annealing temperature. These shape recovery ratios are significantly higher than that of the solutionized specimen at 4% prestrain. The shape recovery ratios decreased with prestrain increase except at 10% prestrain for the specimens annealed at 750 °C and 850 °C. Figure 8b shows the variation of shape recovery strain with prestrain. It seems that the influence of annealing temperature on recovery strain is not significant, although the 650 °C annealing temperature shows a slightly better effect. The recovery strains can increase from approximately 1.5% to 3.8% as the prestrain increases from 4% to 10%, which is higher than that of the solutionized specimen at the same prestrain. 

Compared with the solutionized specimen, the shape recovery ratio and recovery strain showed 61% and 24% increase, respectively, at 4% prestrain for the specimen shot peened and subsequently annealed at 650 °C. These increments attained 67% and 44%, respectively, when the prestrain was increased to 8%. The measured results show that the process of shot peening and subsequent annealing significantly improves the shape memory effect of the Fe-24Mn-6Si-9Cr-6Ni alloy. Severe plastic deformation occurred in the surface layer when the alloys were subjected to shot peening. Stress-induced martensitic transformation, twinning, and dislocation slip contributed to the plastic deformation. Therefore, reverse martensitic transformation, recovery, recrystallization, and even secondary recrystallization could happen during annealing. In this process, a fine microstructure can be formed in the surface layer, as shown in Figure 5 and Figure 6a. It has been observed that the grain refinement can improve the shape memory effect because the grain boundaries can strengthen the parent phase and hinder the growth of martensite in different orientations [28]. Additionally, the α′-martensite was introduced during shot peening and remained after annealing in this study. The presence of α′-martensite is assumed to decrease the permanent slip during prestraining due to its higher yield strength compared with that of the γ-austenite; thus, a higher degree of shape recovery can be obtained [27]. Moreover, research has shown that the α′-martensite introduced using thermomechanical treatment can prevent collisions between different ε-martensite bands and make the bands form in a domain-specific manner during deformation, benefiting the shape memory effect [25].

This study used shot peening and subsequent annealing to process the Fe-24Mn-6Si-9Cr-6Ni alloy. Microstructure refinement and α′-martensite are observed in the specimens after the shot peening and subsequent annealing. Their shape recovery ratio and recovery strain improve significantly compared with those of the solutionized specimen. The results show that shot peening is a potential technology to improve the shape memory effect of Fe-Mn-Si-Cr-Ni alloys. However, the detailed mechanism of the microstructure evolution, phase transformation, and improvement of shape memory effect remains unclear, requiring further research.

## 4. Conclusions

(1) The α′-martensite was introduced in the Fe-24Mn-6Si-9Cr-6Ni alloy during shot peening and remained after annealing. The amount of α′-martensite of the surface layer increased when the annealing temperature increased from 650 °C to 850 °C.
(2) Microstructure of the surface layer was refined after shot peening and subsequent annealing. The amount of recrystallization of the surface layer increased with annealing temperature.
(3) Compared with those of the solutionized specimen, the shape recovery ratio and recovery strain are significantly increased for the Fe-24Mn-6Si-9Cr-6Ni alloy after shot peening and subsequent annealing.

## Figures and Tables

**Table 1:** Alloy chemical composition (in wt%).

Mn	Si	Cr	Ni	Fe
24.22	5.50	9.02	6.06	Balance

**Table 2:** Shot peening parameters.

Shot Material	Shot Size (mm)	Air Pressure (MPa)	Shot Distance (mm)	Almen Intensity	Coverage Ratio (%)
Cast steel	0.3	0.43	150	0.25 A	150

**Table 3:** Crystal feature of the major phases present in Fe-Mn-Si-Cr-Ni alloys.

Phase	Crystal Structure	Lattice Parameters (Å)
γ-austenite	Face-centered cubic	3.588 ≤ a ≤ 3.615
ε-martensite	Hexagonal close-packed	3.5325 ≤ a ≤ 3.5550, 4.0637 ≤ c ≤ 4.1461
α′-martensite	Body-centered cubic	2.86 ≤ a ≤ 2.88

## Data Availability

Not applicable.
